# Multi-PheWAS intersection approach to identify sex differences across comorbidities in 59 140 pediatric patients with autism spectrum disorder

**DOI:** 10.1093/jamia/ocab144

**Published:** 2021-08-18

**Authors:** Alba Gutiérrez-Sacristán, Carlos Sáez, Carlos De Niz, Niloofar Jalali, Thomas N DeSain, Ranjay Kumar, Joany M Zachariasse, Kathe P Fox, Nathan Palmer, Isaac Kohane, Paul Avillach

**Affiliations:** 1 Department of Biomedical Informatics, Harvard Medical School, Boston, Massachusetts, USA; 2 Biomedical Data Science Lab, Instituto Universitario de Tecnologías de la Información y Comunicaciones, Universitat Politècnica de València, Valencia, Spain; 3 Department of General Paediatrics, Erasmus MC Sophia Children's Hospital, University Medical Center Rotterdam, Rotterdam, the Netherlands; 4 Computational Health Informatics Program, Boston Children's Hospital, Boston, Massachusetts, USA

**Keywords:** autism spectrum disorder, sex characteristics, comorbidity, large-scale

## Abstract

**Objective:**

To identify differences related to sex and define autism spectrum disorder (ASD) comorbidities female-enriched through a comprehensive multi-PheWAS intersection approach on big, real-world data. Although sex difference is a consistent and recognized feature of ASD, additional clinical correlates could help to identify potential disease subgroups, based on sex and age.

**Materials and Methods:**

We performed a systematic comorbidity analysis on 1860 groups of comorbidities exploring all spectrum of known disease, in 59 140 individuals (11 440 females) with ASD from 4 age groups. We explored ASD sex differences in 2 independent real-world datasets, across all potential comorbidities by comparing (1) females with ASD vs males with ASD and (2) females with ASD vs females without ASD.

**Results:**

We identified 27 different comorbidities that appeared significantly more frequently in females with ASD. The comorbidities were mostly neurological (eg, epilepsy, odds ratio [OR] > 1.8, 3-18 years of age), congenital (eg, chromosomal anomalies, OR > 2, 3-18 years of age), and mental disorders (eg, intellectual disability, OR > 1.7, 6-18 years of age). Novel comorbidities included endocrine metabolic diseases (eg, failure to thrive, OR = 2.5, ages 0-2), digestive disorders (gastroesophageal reflux disease: OR = 1.7, 6-11 years of age; and constipation: OR > 1.6, 3-11 years of age), and sense organs (strabismus: OR > 1.8, 3-18 years of age).

**Discussion:**

A multi-PheWAS intersection approach on real-world data as presented in this study uniquely contributes to the growing body of research regarding sex-based comorbidity analysis in ASD population.

**Conclusions:**

Our findings provide insights into female-enriched ASD comorbidities that are potentially important in diagnosis, as well as the identification of distinct comorbidity patterns influencing anticipatory treatment or referrals. The code is publicly available (https://github.com/hms-dbmi/sexDifferenceInASD).

## INTRODUCTION

One of the most consistent features reported for autism spectrum disorders (ASDs) has been the imbalance of sex ratio.^1–3^ The current sex ratio is estimated to be 3:1 male-to-female,^4^^,^^5^ varying according to the age and population under study. The reasons for this disproportionate sex imbalance, however, remain unclear. Various biological theories have been proposed, including those related to genes, hormones, and brain structure.^2^^,^^6^ The female protective effect theory^7^^,^^8^ suggests that females require a higher number of genetic mutations to develop ASD, but then present greater ASD severity.^9^^,^^10^ By contrast, males with ASD present a higher genetic variability and decreased ASD severity.^11^ Other studies have identified sex chromosomes as potential risk factors for neurodevelopmental disorders, such as ASD. Some have argued that possession of a second X chromosome might be protective for females.^12^ An additional hypothesis postulates that sexually dimorphic hormonal exposures,^13^^,^^14^ such as high testosterone levels during pregnancy, could account for the preponderance of ASD in males.^15^

While all these hypotheses point to potential mechanisms associated with ASD sex differences, biology is not the only potential explanation for the sexual dimorphism. The development of instruments to detect ASD relied mainly on male samples,^7^^,^^8^ contributing to a possible misdiagnosis of females, late diagnosis when compared with males, or a lack of diagnosis overall.^9–12^ The working hypothesis is that some key features for identifying males with ASD are not as prominent in females,^13–17^ and these cues are often overlooked during diagnosis. Therefore, applying the same ASD diagnostic criteria to both sexes^12^^,^^18^^,^^19^ might have inflated the male-to-female sex ratio.

To study these and other causes further, researchers have conducted analyses of comorbidity patterns. One widely used approach is the phenome-wide association analysis (PheWAS). This approach consists of comparing 2 groups, cases and controls, to identify statistically significant phenotypes more likely to be present in cases than in controls. Cases and controls are defined by a unique variable (eg, having or not a disease). These are useful and arguably critical to unlocking some of the complexities of the disorder, as much of the diversity seen in ASD relates to co-occurring conditions. In addition, the phenotypes that co-occur with ASD may help distinguish distinct ASD subgroups, which, upon thorough investigation, could enhance understanding of the disease. Evidence shows that the comorbidity burden, defined as the coexistence of 2 or more diseases in the same person,^20^ is higher in individuals diagnosed with ASD, as compared with the general population,^21^^,^^22^ including gastrointestinal conditions,^23–25^ epilepsy,^30^ and psychiatric comorbidities.^21^^,^^25^^,^^32^ Yet, very few analyses have examined whether these comorbidity patterns in patients with ASD are distinct for each sex^20–24^ and they have been focused on a predefined list of comorbidities. In addition, there is a dearth of comorbidity research in some age groups, including infants with ASD.^20–24^ Further, investigators have been hindered by insufficient sample size.^13^^,^^20^^,^^21^^,^^25^

Larger, diverse, and more representative samples are required to draw meaningful conclusions about differences between ASD subgroups. We sought to obtain such a sample through the use of real-world data, defined as data relating to patient health status and/or the delivery of health care routinely collected from a variety of sources. We drew on 2 such independent real-world datasets to yield a sizable enough population of individuals with ASD to perform studies that would yield clinically meaningful results.

The primary objective of this study is to develop an approach, the multi-PheWAS intersection, that enable investigators to explore sex differences in real-world datasets, across all diagnostic codes and potential comorbidities. We apply this method to identify female ASD characteristic phenotypes by comparing (1) females with ASD vs males with ASD and (2) females with ASD vs females without ASD. We use another dataset collected from a pediatric university hospital to further validate those results .

The biomedical informatics contributions of this study include (1) the design, implementation of a multi-PheWAS intersection pipeline; (2) reducing the potential bias in the identification of mental disorder patients when using real-world data by increasing the specificity using a threshold of 3 diagnostic codes per patient; and (3) making the full code available in GitHub to enable the reproduction of these results to anyone with access to the datasets analyzed in this study and that can also be easily adapted to other datasets and diseases changing the value of the input parameters.

## MATERIALS AND METHODS

### Data

We analyzed 2 independent real-world datasets: Aetna,^26^ a nationwide claims database of 67 million patients, collected between January 1, 2008, and December 1, 2019, and an independent set of data from 1.7 million patients at Boston Children’s Hospital, collected between January 1, 2008, and September 19, 2016.

**Figure 1.**
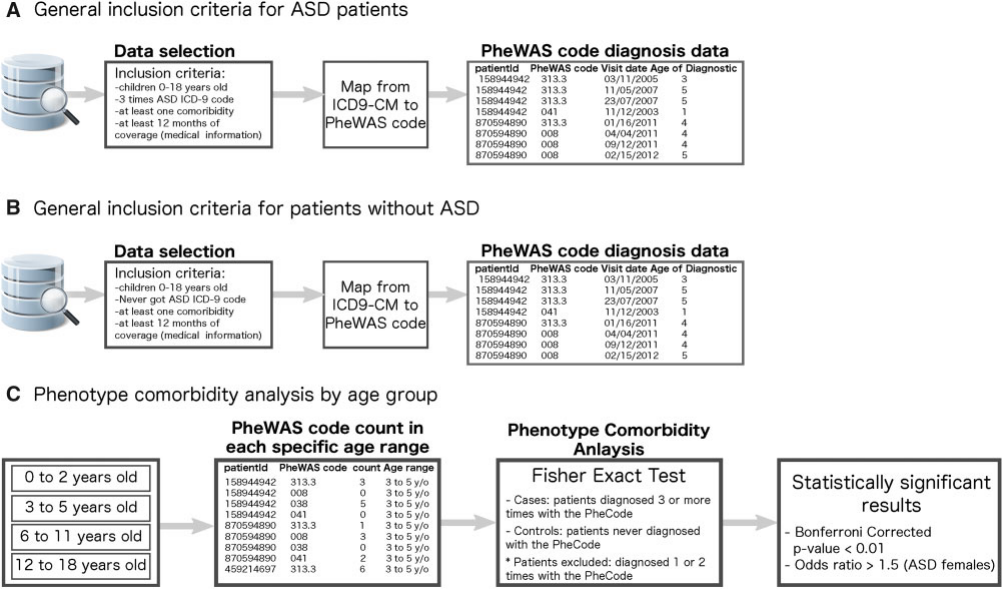
General workflow for the phenotype comorbidity analysis. For our method, we began with our selection of patient data. (A) Autism spectrum disorder (ASD) patient inclusion criteria: 0-18 years of age, assigned an ASD International Classification of Diseases–Ninth Revision–Clinical Modification (ICD-9-CM) code at least 3 times, and records collected for at least 12 months. (B) Patients without ASD inclusion criteria: 0-18 years of age, never assigned an ASD ICD-9-CM code, and records collected for at least 12 months. We then proceeded through our (C) phenotype comorbidity analysis. For each of the phenome-wide association analysis (PheWAS) codes present per set, we ran a Fisher exact test, comparing patients with or without ASD, males vs females.

To be eligible for this study (Figure 1A), individuals 0 to 18 years of age had to be diagnosed upon at least 3 different occasions, based on an International Classification of Diseases–Ninth Revision–Clinical Modification (ICD-9-CM)^27^ code for ASD (299.00, 299.01, 299.80, 299.81, 299.90, 299.91). Eligible children also had at least 1 phenotypic category in addition to ASD and their records collected for more than 12 months (Figure 1B). As a control, we studied all 7 million children without ASD (3 763 418 females and 3 858 927 males) presented in the claims database. To avoid overlap between the 2 datasets, patients from Boston Children’s Hospital were not eligible for this study if their insurance was the same as the claim database.

### Phenotype comorbidity analysis

**Figure 2.**
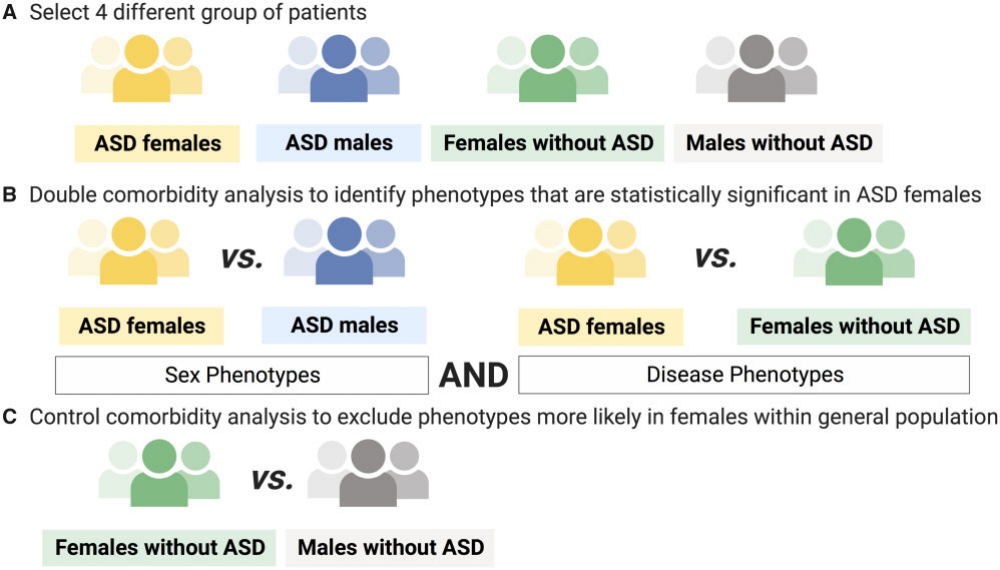
Group patient selection and comparisons: (A) Population stratification in 4 groups. (B) Two comparisons to identify phenotypic codes that were statistically significant in females with autism spectrum disorder (ASD). (C) Control comparison to identify phenotypic codes that were statistically significant in females within the general population, to be excluded from the results. Created with BioRender.com.

To identify statistically significant phenotypic codes that were more likely present in females with ASD, we deployed a multi-PheWAS intersection approach, analyzing the intersection of phenotypes that are significantly different in distinct phenome-wide comparisons (Figure 1C). We first selected and classified 2 groups of patients: cases (females with ASD) vs controls (males with ASD and females without ASD) (Figure 2A). We then systematically examined each phenotypic category to find associations between either sex (females with ASD vs males with ASD) or disease (females with ASD vs females without ASD) (Figure 2B). To identify statistically significant phenotypic codes that were more likely present in females without ASD, we deployed the same phenotype comorbidity approach, comparing females without ASD with males without ASD (Figure 2C). This allows us to identify comorbidities more likely in females in the general population. We then excluded these phenotypic codes from our results because they are not associated with the fact of having ASD.

Diagnoses were initially presented as ICD-9-CM codes (10 279 in the claims and 8585 in the hospital datasets). We aggregated them using PheWAS^28^ codes (1860 in the claims and 1709 in hospital datasets). We then determined the counts for each patient and phenotypic category. To increase the reliability of the reuse of diagnostic codes as a proxy for clinical diagnoses, we restricted our cohort to patients that received ASD diagnostic codes at least 3 distinct times.^28^ The same approach was used for the comorbid diagnosis, only those recorded on at least 3 distinct occasions were included in our analysis.

We analyzed all phenotypic codes presented by patients as follows: phenotypic codes diagnosed in patients 0 to 2 years of age (infants), 3 to 5 years of age (prepubertal), 6 to 11 years of age (peripubertal), and 12 to 18 years of age (postpubertal).^29^ Each patient can contribute across several age groups with different phenotypes during their development. For each phenotypic code and age group, we ran a Fisher exact test, based on diagnosis or lack of diagnosis with ASD, and separately, sex. We estimated the confidence interval (CI), odds ratio (OR), and *P* value. The conservative Bonferroni correction was applied to correct for multiple testing. To be considered more likely to occur in females with ASD, a phenotypic code had to be statistically significant with a corrected *P* value <.01/total. Phenotype codes at each age group (detailed information in Supplementary Material 1), as measured by comorbidity testing, and have an OR >1.5 (meaning 1.5× more likely to be present in one group as compared with other groups). This cutoff of at least 1.5 was applied to get closer to results that could potentially be clinically significant. We explored how many statistically significant results were obtained from multiple OR cutoffs from 1.1 to 2.0. For visualization purposes, the heatmaps have been organized based on the phenotype category (eg, “Epilepsy,” “Partial epilepsy,” and “Convulsions” are plotted in consecutive rows as they all belong to the category “Neurological”). These higher phenotype categories have been extracted from the phecode definition file available at the PheWAS catalog site (https://phewascatalog.org/phecodes_v1_1). The analysis was performed using SQL and R version 3.4.1 (R Foundation for Statistical Computing, Vienna, Austria). Open-source code is publicly available at GitHub (https://github.com/hms-dbmi/sexDifferenceInASD).

The study was approved by the central Institutional Review Board at the National Human Genome Research Institute (IRB-P00026337).

## RESULTS

After the inclusion criteria, using claims data, we analyzed 59 140 individuals with ASD (11 440 females and 47 700 males) that met our inclusion criteria. We further validated our findings using an independent pediatric hospital dataset, composed of 3728 patients with ASD (762 females and 2966 males) and 28 740 randomly selected individuals without ASD (13 988 females and 14 752 males) after applying the inclusion criteria.

### Identification of phenotypes in the claims dataset

**Figure 3.**
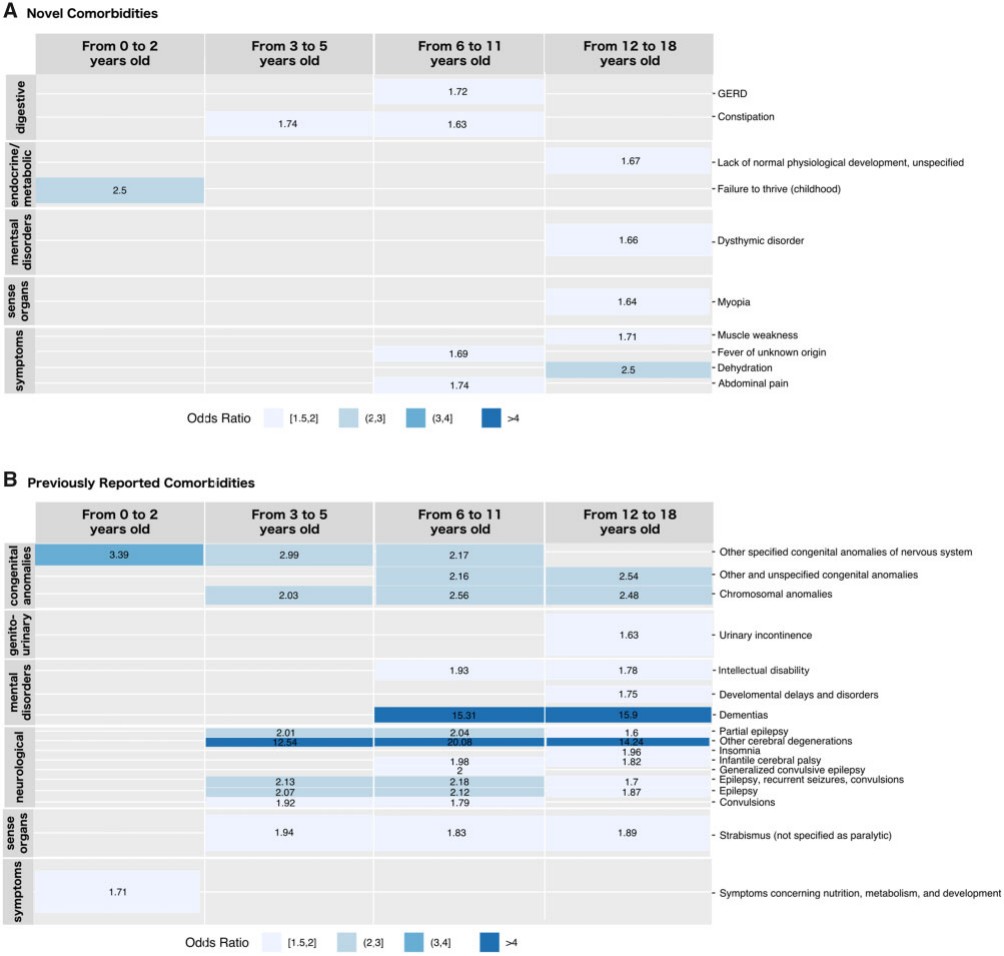
Comorbidities more likely to appear in females with autism spectrum disorder (ASD) in the claims database by age groups: (A) novel comorbidities and (B) previously reported comorbidities. The y-axis on the left represents the phenotype categories (added for visualization purposes to sort the phecodes on the right in a meaningful way), the y-axis on the right represent the specific phenotype codes (phecodes). The x-axis represents the 4 different age groups. The cell color is related to the odds ratio when comparing females with ASD vs males with ASD. GERD: gastroesophageal reflux disease.

We discovered 27 distinct phenotype codes that were more likely present in females with ASD, as compared with males with ASD and females without ASD (Table 1, Figure 3, and Supplementary Tables S1 and S2, which contains the raw numbers of females and males with and without ASD for each specific phenotype category). Demonstrating specificity to ASD, we showed that these phenotype codes were not more likely present in females without ASD, as compared with males without ASD (Supplementary Table S3). Supplementary Table S3 does not contain all the phenotypes that were more likely in females without ASD vs males without ASD, it only contains those phenotypes that were also significantly more prevalent in females with ASD in the other 2 compared groups.

**Table 1.** Phenotypic codes that were significantly more likely to present in females with ASD in the claims database, by age group

Age group	Phenotype code	Female vs male		ASD vs non ASD	
		OR	95% CI	OR	95% CI
0-2 y	Other specified congenital anomalies of nervous system	3.386	2.048-5.588	12.016	8.258-16.941
0-2 y	Failure to thrive (childhood)	2.499	1.807-3.438	4.498	3.484-5.724
0-2 y	Symptoms concerning nutrition, metabolism, and development	1.708	1.375-2.115	4.74	3.949-5.652
3-5 y	Other cerebral degenerations	12.543	5.481-32.206	72.042	44.257-113.241
3-5 y	Other specified congenital anomalies of nervous system	2.991	2.109-4.223	24.262	18.309-31.617
3-5 y	Epilepsy, recurrent seizures, convulsions	2.126	1.653-2.719	34.544	27.822-42.634
3-5 y	Epilepsy	2.07	1.542-2.759	32.671	25.301-41.628
3-5 y	Chromosomal anomalies	2.033	1.544-2.66	15.01	11.892-18.731
3-5 y	Partial epilepsy	2.013	1.518-2.65	31.128	24.352-39.279
3-5 y	Strabismus (not specified as paralytic)	1.936	1.586-2.357	4.958	4.192-5.829
3-5 y	Convulsions	1.916	1.618-2.263	22.899	19.772-26.377
3-5 y	Constipation	1.744	1.463-2.071	6.715	5.78-7.765
6-11 y	Other cerebral degenerations	20.083	8.734-53.75	50.25	33.36-73.628
6-11 y	Dementias	15.307	5.476-52.777	120.02	64.072-217.684
6-11 y	Chromosomal anomalies	2.562	2.144-3.055	25.225	21.715-29.167
6-11 y	Epilepsy, recurrent seizures, convulsions	2.178	1.86-2.545	33.37	29.055-38.152
6-11 y	Other specified congenital anomalies of nervous system	2.174	1.666-2.821	24.521	19.482-30.516
6-11 y	Other and unspecified congenital anomalies	2.163	1.614-2.877	18.506	14.415-23.439
6-11 y	Epilepsy	2.122	1.749-2.566	23.497	19.877-27.574
6-11 y	Partial epilepsy	2.037	1.726-2.397	27.776	24.032-31.934
6-11 y	Generalized convulsive epilepsy	1.999	1.581-2.516	31.497	25.573-38.416
6-11 y	Infantile cerebral palsy	1.983	1.611-2.432	16.813	14.056-19.978
6-11 y	Intellectual disability	1.934	1.651-2.259	131.481	111.943-153.279
6-11 y	Strabismus (not specified as paralytic)	1.834	1.599-2.1	5.306	4.718-5.948
6-11 y	Convulsions	1.791	1.589-2.017	23.617	21.217-26.182
6-11 y	Abdominal pain	1.741	1.525-1.985	2.056	1.835-2.297
6-11 y	GERD	1.721	1.436-2.056	7.853	6.715-9.133
6-11 y	Fever of unknown origin	1.69	1.489-1.916	2.505	2.245-2.787
6-11 y	Constipation	1.632	1.448-1.838	7.785	7.012-8.625
12-18 y	Dementias	15.899	4.289-87.753	82.438	39.601-158.28
12-18 y	Other cerebral degenerations	14.238	5.984-39.011	54.069	33.839-83.481
12-18 y	Other and unspecified congenital anomalies	2.542	1.867-3.444	28.54	22.088-36.367
12-18 y	Dehydration	2.499	1.743-3.554	4.604	3.448-6.03
12-18 y	Chromosomal anomalies	2.482	2.009-3.059	27.246	22.863-32.274
12-18 y	Insomnia	1.964	1.499-2.556	10.151	8.087-12.587
12-18 y	Strabismus (not specified as paralytic)	1.894	1.539-2.323	8.487	7.122-10.042
12-18 y	Epilepsy	1.866	1.51-2.298	25.823	21.486-30.794
12-18 y	Infantile cerebral palsy	1.817	1.456-2.257	21.134	17.458-25.391
12-18 y	Intellectual disability	1.783	1.568-2.023	162.89	141.49-184.347
12-18 y	Developmental delays and disorders	1.745	1.558-1.952	76.338	68.867-84.751
12-18 y	Muscle weakness	1.706	1.377-2.105	3.355	2.797-3.994
12-18 y	Epilepsy, recurrent seizures, convulsions	1.696	1.455-1.973	30.268	26.447-34.559
12-18 y	Lack of normal physiological development, unspecified	1.671	1.404-1.983	71.896	61.121-83.963
12-18 y	Dysthymic disorder	1.659	1.393-1.971	4.532	3.903-5.236
12-18 y	Myopia	1.637	1.325-2.014	1.765	1.474-2.098
12-18 y	Urinary incontinence	1.627	1.376-1.918	24.359	21.014-28.085
12-18 y	Partial epilepsy	1.598	1.349-1.887	28.837	24.812-33.374

Phenotypic codes that were statistically significant (corrected *P* value < .01) and more likely to appear in females with ASD, as compared with males with ASD and females without ASD (OR >1.5). For each phenotypic code, we presented the OR and the 95% CI (based on a comparison between females and males with ASD and females with or without ASD). Phenotypic codes present in this table were not statistically significant in females without ASD in the general population. The table is sorted according to female vs male OR in decreasing order.

ASD: autism spectrum disorder; CI: confidence interval; GERD: gastroesophageal reflux disease; OR: odds ratio.

We found 3 phenotypic codes shared in the infant group, 9 in prepubertal, 17 in peripubertal, and 18 in adolescence (Table 1). The 3 phenotypic infant codes were other specified congenital anomalies of the nervous system, failure to thrive, and symptoms related to nutrition, metabolism, and development (ASD female and ASD male: ORs of 3.3 [95% CI, 2.048-5.588], 2.5 [95% CI, 1.807-3.438], and 1.7 [95% CI, 1.375-2.115], respectively). The same codes had ORs of 12 (95% CI, 8.258-16.941), 4.5 (95% CI, 3.484-5.724), and 4.7 (95% CI, 3.949-5.652), respectively, when comparing females with and without ASD. For those diagnosed in prepubertal, 5 of 9 phenotypic codes identified were neurological, 2 were congenital, 1 was digestive (constipation with ASD female and ASD male : OR, 1.74 [95% CI, 1.463-2.071]; and with ASD female and non-ASD female: OR, 6.7 [95% CI, 5.78-7.765]), and 1 was sense organs (strabismus not specified as paralytic with an ASD female and ASD male: OR, 1.94 [95% CI, 1.586-2.357]; and with an ASD female and non-ASD female: OR, 4.5 [95% CI, 4.192-5.829]).

For individuals diagnosed in peripubertal, we discovered 17 phenotypic codes that more likely appeared in females with ASD. As with prepubertal, most phenotypic codes in the peripubertal group were neurological and congenital. Digestive issues (gastroesophageal reflux disease and constipation) as well as sense organs (strabismus) were also found in this age group as more likely to occur in females with ASD. When comparing females and males with ASD in the same age group, the phenotypic code with the highest OR of 20.1 (95% CI, 8.734-53.75) was “other cerebral degenerations.” By contrast, when comparing females with and without ASD, the phenotypic code that demonstrated the highest OR of 131 (95% CI, 111.943-153.279) was intellectual disability.

For diagnosis in adolescence, we found 18 phenotypic codes, most of them of neurological and psychiatric. Of these, 10 were also reported in other age groups. Digestive issues were not more likely to occur in females with ASD in this age group, while lack of normal physiological development unspecified (ASD female and ASD male: OR, 1.67 [95% CI, 1.404-1.983]; and ASD female and non-ASD female: OR, 71.9 [95% CI, 61.121-83.963]), as well as urinary incontinence (ASD female and ASD male: OR, 1.6 [95% CI, 1.376-1.918] and ASD female and non-ASD female: OR, 24.3 [95% CI, 21.014-28.085]), muscle weakness (ASD female and ASD male: OR, 1.7 [95% CI, 1.377-2.105]; and ASD female and non-ASD female: OR, 3.3 [95% CI, 2.797-3.994]), and myopia (ASD female and ASD male: OR, 1.6 [95% CI, 1.325-2.014]; and ASD female and non-ASD female: OR, 1.7 [95% CI, 1.474-2.098]), appeared for the first time in this age group.

When comparing phenotypic codes that were nonspecific to age (Figure 3), we found 6 phenotypic codes shared by children with ASD between 3 and 18 years of age (strabismus, other cerebral degenerations, chromosomal anomalies, and epilepsy-related disorders). Specifically, strabismus (mostly esotropia, exotropia not otherwise specified, and accommodative component in esotropia) was more likely to occur in females with ASD when compared with females without ASD (in patients 3-5 years of age: OR, 4.9 [95% CI, 4.192-5.829]; in patients 6-11 years of age: OR, 5.3 [95% CI, 4.718-5.948]; and in patients 12-18 years of age: OR, 8.4 [95% CI, 7.122-10.042]) and with an OR close to 1.9 when compared with males with ASD. Epilepsy was more likely to occur in females with ASD (ASD females and non-ASD females: OR, 32.6 [95% CI, 25.301-41.628] in patients 3-5 years of age; OR, 23.5 [95% CI, 19.877-27.574] in patients 6-11 years of age; and OR, 25.8 [95% CI, 21.486-30.794] in patients 12-18 years of age; ASD female and ASD male: OR, 2 [95% CI, 1.542-2.759] in patients 3-5 years of age; OR, 2.1 [95% CI, 1.749-2.566] in patients 6-11 years of age; and OR, 1.8 [95% CI, 1.51-2.298] in patients 12-18 years of age).

### Identification of phenotypes in the pediatric hospital dataset

Using the pediatric hospital dataset, we detected 7 phenotypic codes that were more likely to be present in females with ASD (Table 2, Supplementary Figure S2, Tables S4 and S5). Of those, 6 were identical to the ones found in the claims data, specifically chromosomal anomalies, developmental delays and disorders, other cerebral degenerations, epilepsy, recurrent seizures and convulsions, and epilepsy and convulsions. These 6 phenotypic codes were found as statistically significant only in the 6- to 11-years-old group. Only one phenotypic code milestone was specific to the pediatric hospital dataset, and it was only associated with sex dimorphism in patients 3 to 5 years of age.

**Table 2.** Phenotypic codes that were significantly more likely to be present in females with ASD in the pediatric hospital dataset, by age group

Age group	Phenotype code	Female vs male		ASD vs non ASD	
		OR	95% CI	OR	95% CI
3-5 y	Delayed milestones	3.458	2.029-5.859	8.907	5.563-13.943
6-11 y	Other cerebral degenerations	14.035	4.387-58.8	9.824	4.734-19.525
6-11 y	Epilepsy	3.363	2.196-5.125	5.306	3.69-7.501
6-11 y	Chromosomal anomalies	2.32	1.698-3.151	5.86	4.408-7.719
6-11 y	Epilepsy, recurrent seizures, convulsions	2.263	1.577-3.219	5.425	3.906-7.433
6-11 y	Convulsions	2.19	1.61-2.961	5.173	3.915-6.771
6-11 y	Developmental delays and disorders	1.694	1.357-2.111	10.984	8.886-13.56

Phenotypic codes that were statistically significant (corrected *P* value < .01) and more likely to appear in females with ASD, as compared with males with ASD, and males and females without ASD (OR > 1.5). For each phenotypic code, we presented the OR and the 95% CI (based on the comparison between females and males with ASD and females with or without ASD). Phenotypic codes present in this table were not statistically significant in females without ASD in the general population. The table is sorted according to female vs male OR in decreasing order.

ASD: autism spectrum disorder; CI: confidence interval; OR: odds ratio.

## DISCUSSION

In this study, we applied the multi-PheWAS intersection approach in a large-scale, real-world claims dataset and we identified 27 phenotypic codes more likely present in females with ASD, 10 not previously reported. We further validated our findings using an independent pediatric hospital dataset. With this approach, we overcame one of the main limitations experienced in the field of ASD research: population sample size for females.^20^^,^^21^ The strength of this approach was further enhanced by the performance of a systematic and large-scale analysis of comorbidity patterns. We were able to analyze all possible ASD comorbidities at the PheWAS level, without having to reduce the analysis to a specific set.

Our results support prior findings. We showed that neurological disorders, such as epilepsy, were more likely to occur in females with ASD, as compared with females without ASD and with males with ASD, in all children 3 to 18 years of age. Epilepsy was also significantly prevalent in the pediatric hospital dataset for patients 6 to 11 years of age. Supekar et al^23^ and Amiet et al^30^ also reported a higher prevalence of epilepsy in females due to the increased prevalence of cognitive and motor deficits. These results align with our analysis: we found the prevalence of epilepsy and intellectual disabilities to be twice as high in females with ASD compared with males with ASD for groups 3 to 18 years of age (Supplementary Table S6). Notably, females and males without ASD exhibited the same prevalence of the 2 comorbidities, between 0.03% and 0.04%. Our study also reported muscle weakness to be more prevalent in females without ASD (OR, 1.7 [95% CI, 1.37-2.10]), confirming Ben-Itzchak's study,^33^ in which musculoskeletal deficits were significantly higher overall in females (73.8%) than in males (57.1%).

Two other comorbidities have been previously reported as being more prevalent in females with ASD compared with males: urinary incontinence (OR, 1.627 [95% CI, 1.376-1.918] in ASD females and ASD males; and OR, 24.3 [95% CI, 21.014-28.085] in ASD females and non-ASD females, 12-18 years of age) and strabismus (OR >1.8 in ASD females and ASD males 3-18 years of age; OR, 4.9 [95% CI, 4.718-5.948] in ASD females and non-ASD females 3-5 years of age; OR, 5.3 [95% CI, 4.718-5.948] in ASD females and non-ASD females 6-11 years of age; OR, 8.5 [95% CI, 7.122-10.042] in ASD females and non-ASD females 12-18 years of age). However, the underlying reason for the discrepancy was the use of risperidone for urinary incontinence^31^ and the use of valproate for strabismus. In the claims dataset, none of the patients with strabismus were treated with valproate, and for the individuals with urinary incontinence, a higher percentage of males (17.49%) than females (14.08%) with ASD were treated with risperidone. Other underlying causes could explain the higher number of females with ASD that show these 2 comorbidities.

Davignon et al^32^ analyzed comorbidities within a dataset of 4123 individuals with ASD and more than 1000 distinct ICD-9 codes, grouped into 175 conditions. However, the study did not focus on a pediatric population. Other ASD comorbidity studies had a small sample size and did not factor in sex differences. They were also restricted to a predefined comorbidity list, derived mainly from the most well-known ASD comorbidities.^20^^,^^23^^,^^25^ Finally, other studies have focused only on one comorbidity: suicidal ideation^34^ or epilepsy.^23^^,^^30^ By contrast, our study simultaneously targeted all existing comorbidities without relying on a predefined list of ASD comorbidities.

Beyond supporting prior findings, our comorbidity analysis further uncovered evidence of 10 novel phenotypes—gastroesophageal reflux disease; constipation; lack of normal physiological development; failure to thrive; dysthymic disorder; myopia; symptoms concerning nutrition, metabolism, and development; fever of unknown origin; dehydration; and abdominal pain. While known to be associated with ASD, these were not previously associated with sex dimorphism in ASD. These results could be explained by selection bias: females are only diagnosed when they have more severe symptoms, that is, epilepsy, owing to underlying pathology in females. Still, the signs and symptoms that we identified likely signal underlying common pathways and the need for further study.

When we compared the 2 datasets, claims and pediatric hospital, we found that for the age group 6 to 11 years old, they shared 6 of the 17 comorbidities first identified in the claim data. Out of the 7 comorbidities found in the pediatric hospital, 6 were identified in the claim dataset. The one present in the pediatric hospital but not in the claims dataset, “Delayed milestones” in the age group 3 to 5 years old, might be explained by the distinct type of claims data we used. Boston Children’s Hospital treats a disproportionate number of patients with complex and rare conditions. By contrast, the nationwide claim database contains patients from a more representative and national population. The high concentration of complexity in the overall pediatric hospital dataset patient population could have easily contributed to the differences appearing between datasets in this study.

In summary, sex an important specifier that could help in defining ASD subgroups, identifying mechanisms for disease and potential shared druggable pathways. This systematic, large-scale analysis of sex differences between comorbidities presented in individuals with ASD addresses the skewed sex ratio historically reported in ASD. Results support the notion that the apparent sex difference can be explained in part by differences in the nature of the phenotypic codes that manifest in males vs females with ASD. By understanding those phenotypes more likely present in females, ASD diagnosis in females may be improved. Such phenotypic categorization could help clinicians in guidance and specialist referrals appropriate for the specific anticipated comorbidities. Moreover, as clinicians account for sex as an important variable, it might aid in the identification of distinct comorbidity patterns that could define new ASD subtypes and the best course of treatment. These additional clinical correlates could also help to develop clinical decisive support models and to identify potential ASD subgroups.

### Limitations

The sources of data used in this study may have introduced bias. Specifically, ICD-9-CM terminology was designed for billing and administrative purposes and therefore may include coding bias. Even if the sample size would have been bigger if applying less stringent thresholds (109 305 patients with 1 ASD code, 80 374 patients with 2 ASD codes), we preferred to reduce this potential bias by mapping to a greater level of aggregation, using PheWAS codes, and limiting the eligibility of patients to those diagnosed with the same code at least 3 independent times. Using datasets such as claims data allows you to be more restrictive and still have a big sample size . We were interested to show that this is possible. We increased the specificity by decreasing the number of patients. This is important for psychiatric conditions such as autism. Taking as cases only those patients diagnosed on 3 different occasions with the phenotype, we reduce the possibility that, for example, the code was used related to ASD testing, and not necessarily to ASD diagnosis. We would not use the same approach if our target disorder would be an acute one (eg, myocardial infarction). In this case, one code approach would have been robust enough. Patients only diagnosed 1 or 2 times with the phenotype were considered neither cases nor controls, and were excluded from the analysis.

Because we searched for associations without a hypothesis, there is a chance that some findings may involve noise. There also may be systematic bias and potential misclassification in the underlying database. For instance, in our results, dementia appeared in the claims data as a statistically significant phenotypic category of patients younger than 18 years of age (0.001%).

We also assert that claims data and electronic health records have been recognized as excellent sources of information for comorbidity clinical research.^35^ In fact, data repurposing provides an unprecedented opportunity to use healthcare data for additional research purposes. Finally, the alignment of our results with those from previous reports supports the robustness of our method and the validity of our results.

In this study, we have focused on sex differences, understanding sex as the biological sex, not the social construct gender. Recent studies have shown that transgender and gender-diverse individuals have higher rates of autism diagnosis and traits as well as related neurodevelopmental and psychiatric conditions.^36^ Future studies should include gender as a covariate. Although gender is not automatically recorded in claims and electronic health records, gender dysphoria is. In our dataset we did not find statistically significant differences in terms of gender dysphoria when comparing females with ASD and males with ASD, but it is statistically significantly higher in females with ASD than in females without ASD for the age group 12 to 18 years of age. Further studies would be required and in this specific case without aggregating at a higher level. Using the ICD-10 codification, F64-related codes could be used as a starting point when using this medical data. This will broaden the way we think about autism and other disorders, going from the binary idea of just “women” and “men”.

## CONCLUSIONS

The present study uniquely contributes to the growing body of research regarding sex differences in ASD by overcoming one of the main limitations of previous studies: small sample size. Further, we did not focus our analysis a priori on a selected group of specific disorders. Instead, we analyzed all clinical phenotypic codes applying the multi-PheWAS intersection approach on big real-world data, detecting comorbidity patterns in all patients with ASD to uncover potential sex differences. Overall, when looking for ASD subgroups, sex is an important specifier that can ultimately help in defining ASD subgroups and in the identification of novel mechanisms for disease and so advance our understanding of the disorder.

## FUNDING

This work has been supported by the National Institutes of Health BD2K grant U54HG007963. JMZ received grants from Stichting de Drie Lichten and Stichting Sophia Kinderziekenhuis Fonds for a research internship at Harvard Medical School.

## AUTHOR CONTRIBUTIONS

AG-S conceptualized and designed the study, collected the data, carried out the analyses, drafted the initial manuscript, and reviewed and revised the manuscript. CS and CDN provided feedback in the design of the study and statistical methods, and critically reviewed the manuscript for important intellectual content. NJ collected data, carried out the validation analyses, and reviewed and revised the manuscript. RK and TND supervised the data extraction methodology and reviewed and revised the manuscript. KPF, JMZ, NP, and IK provided critical feedback on the methods and results and critically reviewed the final draft for important intellectual content. PA conceptualized and designed the study, coordinated and supervised data collection, and critically reviewed the manuscript for important intellectual content. All authors approved the final manuscript as submitted and agree to be accountable for all aspects of the work.

## SUPPLEMENTARY MATERIAL

Supplementary material is available at *Journal of the American Medical Informatics Association* online .

## CONFLICT OF INTEREST

The autors have no biomedical financial interest or potential conflict of interest to disclose.

## DATA AVAILABILITY STATEMENT

Individual participant data will not be available.

## Supplementary Material

ocab144_Supplementary_File

## References

[1] Halladay AK , Bishop S, Constantino JN, et al Sex and gender differences in autism spectrum disorder: summarizing evidence gaps and identifying emerging areas of priority. Mol Autism 2015; 6: 36. 26075049 10.1186/s13229-015-0019-y PMC4465158

[2] Ferri SL , Abel T, Brodkin ES. Sex differences in autism spectrum disorder: a review. Curr Psychiatry Rep 2018; 20 (2): 9. 29504047 10.1007/s11920-018-0874-2 PMC6477922

[3] Xu G , Strathearn L, Liu B, et al Prevalence and treatment patterns of autism spectrum disorder in the United States, 2016. JAMA Pediatr 2019; 173 (2): 153–9. 30508021 10.1001/jamapediatrics.2018.4208 PMC6439607

[4] Loomes R , Hull L, Mandy WPL. What is the male-to-female ratio in autism spectrum disorder? A systematic review and meta-analysis. J Am Acad Child Adolesc Psychiatry 2017; 56 (6): 466–74. 28545751 10.1016/j.jaac.2017.03.013

[5] Shattuck PT , Durkin M, Maenner M, et al Timing of identification among children with an autism spectrum disorder: findings from a population-based surveillance study. J Am Acad Child Adolesc Psychiatry 2009; 48 (5): 474–83. 19318992 10.1097/CHI.0b013e31819b3848 PMC3188985

[6] Schaafsma SM , Pfaff DW. Etiologies underlying sex differences in autism spectrum disorders. Front Neuroendocrinol 2014; 35 (3): 255–71. 24705124 10.1016/j.yfrne.2014.03.006

[7] Duvekot J , van der Ende J, Verhulst FC, et al Factors influencing the probability of a diagnosis of autism spectrum disorder in girls versus boys. Autism 2017; 21 (6): 646–58. 27940569 10.1177/1362361316672178

[8] Mandy W , Lai M-C. Towards sex- and gender-informed autism research. Autism 2017; 21 (6): 643–5. 28749233 10.1177/1362361317706904

[9] Gould J. Towards understanding the under-recognition of girls and women on the autism spectrum. Autism 2017; 21 (6): 703–5. 28749237 10.1177/1362361317706174

[10] Rutherford M , McKenzie K, Johnson T, et al Gender ratio in a clinical population sample, age of diagnosis and duration of assessment in children and adults with autism spectrum disorder. Autism 2016; 20 (5): 628–34. 26825959 10.1177/1362361315617879

[11] Begeer S , Mandell D, Wijnker-Holmes B, et al Sex differences in the timing of identification among children and adults with autism spectrum disorders. J Autism Dev Disord 2013; 43 (5): 1151–6. 23001766 10.1007/s10803-012-1656-z

[12] Kothari R , Skuse D, Wakefield J, et al Gender differences in the relationship between social communication and emotion recognition. J Am Acad Child Adolesc Psychiatry 2013; 52 (11): 1148–57.e2. 24157389 10.1016/j.jaac.2013.08.006 PMC3989041

[13] Kreiser NL , White SW. ASD traits and co-occurring psychopathology: the moderating role of gender. J Autism Dev Disord 2015; 45 (12): 3932–8. 26324249 10.1007/s10803-015-2580-9

[14] Lai M-C , Lombardo MV, Auyeung B, et al Sex/gender differences and autism: setting the scene for future research. J Am Acad Child Adolesc Psychiatry 2015; 54 (1): 11–24. 25524786 10.1016/j.jaac.2014.10.003 PMC4284309

[15] Ratto AB , Kenworthy L, Yerys BE, et al What about the girls? Sex-based differences in autistic traits and adaptive skills. J Autism Dev Disord 2017; 48 (5): 1698–711*.* 10.1007/s10803-017-3413-9 PMC5925757 29204929

[16] Kreiser NL , White SW. ASD in females: are we overstating the gender difference in diagnosis? Clin Child Fam Psychol Rev 2014; 17 (1): 67–84. 23836119 10.1007/s10567-013-0148-9

[17] Frazier TW , Georgiades S, Bishop SL, et al Behavioral and cognitive characteristics of females and males with autism in the Simons Simplex Collection. J Am Acad Child Adolesc Psychiatry 2014; 53 (3): 329–40.e1–3. 24565360 10.1016/j.jaac.2013.12.004 PMC3935179

[18] Beggiato A , Peyre H, Maruani A, et al Gender differences in autism spectrum disorders: divergence among specific core symptoms. Autism Res 2017; 10 (4): 680–9. 27809408 10.1002/aur.1715

[19] Dworzynski K , Ronald A, Bolton P, et al How different are girls and boys above and below the diagnostic threshold for autism spectrum disorders? J Am Acad Child Adolesc Psychiatry 2012; 51 (8): 788–97. 22840550 10.1016/j.jaac.2012.05.018

[20] Stacy ME , Zablotsky B, Yarger HA, et al Sex differences in co-occurring conditions of children with autism spectrum disorders. Autism 2014; 18 (8): 965–74. 24126865 10.1177/1362361313505719

[21] Hartley SL , Sikora DM. Sex differences in autism spectrum disorder: an examination of developmental functioning, autistic symptoms, and coexisting behavior problems in toddlers. J Autism Dev Disord 2009; 39 (12): 1715–22. 19582563 10.1007/s10803-009-0810-8 PMC3590797

[22] Palmer N , Beam A, Agniel D, et al Association of sex with recurrence of autism spectrum disorder among siblings. JAMA Pediatr 2017; 171 (11): 1107–12. 28973142 10.1001/jamapediatrics.2017.2832 PMC5710368

[23] Supekar K , Iyer T, Menon V. The influence of sex and age on prevalence rates of comorbid conditions in autism. Autism Res 2017; 10 (5): 778–89. 28188687 10.1002/aur.1741

[24] Doshi-Velez F , Ge Y, Kohane I. Comorbidity clusters in autism spectrum disorders: an electronic health record time-series analysis. Pediatrics 2014; 133 (1): e54–63. 24323995 10.1542/peds.2013-0819 PMC3876178

[25] Brookman-Frazee L , Stadnick N, Chlebowski C, et al Characterizing psychiatric comorbidity in children with autism spectrum disorder receiving publicly funded mental health services. Autism 2018; 22 (8): 938–52. 28914082 10.1177/1362361317712650 PMC6491206

[26] Lakhani CM , Tierney BT, Manrai AK, et al Repurposing large health insurance claims data to estimate genetic and environmental contributions in 560 phenotypes. Nat Genet 2019; 51 (4): 764–5. doi:10.1038/s41588-018-0313-7 10.1038/s41588-019-0377-z 30814726

[27] World Health Organization. International Statistical Classification of Diseases and Related Health Problems. Geneva, Switzerland: World Health Organization; 2004.

[28] Denny JC , Ritchie MD, Basford MA, et al PheWAS: demonstrating the feasibility of a phenome-wide scan to discover gene-disease associations. Bioinformatics 2010; 26 (9): 1205–10. 20335276 10.1093/bioinformatics/btq126 PMC2859132

[29] Williams K , Thomson D, Seto I, et al; StaR Child Health Group. Standard 6: age groups for pediatric trials. Pediatrics 2012; 129 (Suppl 3): S153–60. 22661762 10.1542/peds.2012-0055I

[30] Amiet C , Gourfinkel-An I, Bouzamondo A, et al Epilepsy in autism is associated with intellectual disability and gender: evidence from a meta-analysis. Biol Psychiatry 2008; 64 (7): 577–82. 18565495 10.1016/j.biopsych.2008.04.030

[31] Kumazaki H , Watanabe K, Imasaka Y, et al Risperidone-associated urinary incontinence in patients with autistic disorder with mental retardation. J Clin Psychopharmacol 2014; 34 (5): 624–6. doi:10.1097/jcp.0000000000000197 25118082 10.1097/JCP.0000000000000197

[32] Davignon MN , Qian Y, Massolo M, et al Psychiatric and medical conditions in transition-aged individuals with ASD. Pediatrics 2018; 141 (Suppl 4): S335–45. 29610415 10.1542/peds.2016-4300K

[33] Ben-Itzchak E, , Ben-Shachar S, , Zachor DA. Specific neurological phenotypes in autism spectrum disorders are associated with sex representation. Autism Res 2013; 6 (6): 596–604. 23873852 10.1002/aur.1319

[34] Kirby AV , Bakian AV, Zhang Y, et al A 20-year study of suicide death in a statewide autism population: Suicide and ASD. Autism Res 2019; 86 (4): 658–66. 10.1002/aur.2076 PMC6457664 30663277

[35] Pendergrass SA , Crawford DC. Using electronic health records to generate phenotypes for research. Curr Protoc Hum Genet 2019; 100 (1): e80. 30516347 10.1002/cphg.80 PMC6318047

[36] Warrier V , Greenberg DM, Weir E, et al Elevated rates of autism, other neurodevelopmental and psychiatric diagnoses, and autistic traits in transgender and gender-diverse individuals. Nat Commun 2020; 11 (1): 3959. 32770077 10.1038/s41467-020-17794-1 PMC7415151

