# Supplementary material for: Multi-PheWAS intersection approach to identify sex differences across comorbidities in 59 140 pediatric patients with autism spectrum disorder
Source: J Am Med Inform Assoc. 2021 Aug 18;29(2):230–8. doi: 10.1093/jamia/ocab144 (PMC8757290; doi:10.1093/jamia/ocab144)
Supplement: ocab144_Supplementary_File [file ocab144_supplementary_file.docx]

**Supplemental information**

Additional supplemental information may be found:

- TableS1: Phenotype categories that are statistically significant in the claims database (females with ASD as compared to males with ASD)
- TableS2: Phenotype categories that are statistically significant in the claims database (females with ASD as compared to females without ASD)
- TableS3: Excluded phenotype categories per age group
- FigS1: Comorbidities more likely to appear in females with ASD in the pediatric hospital by age groups.
- TableS4: Phenotype categories that are statistically significant in the pediatric hospital dataset (females with ASD as compared to males with ASD)
- TableS5: Phenotype categories that are statistically significant in the pediatric hospital dataset (females with ASD as compared to females without ASD)
- TableS6: Prevalence of epilepsy and intellectual disabilities in ASD and non-ASD individuals across the four different age groups
- Supplemental 1: Multiple testing correction

*
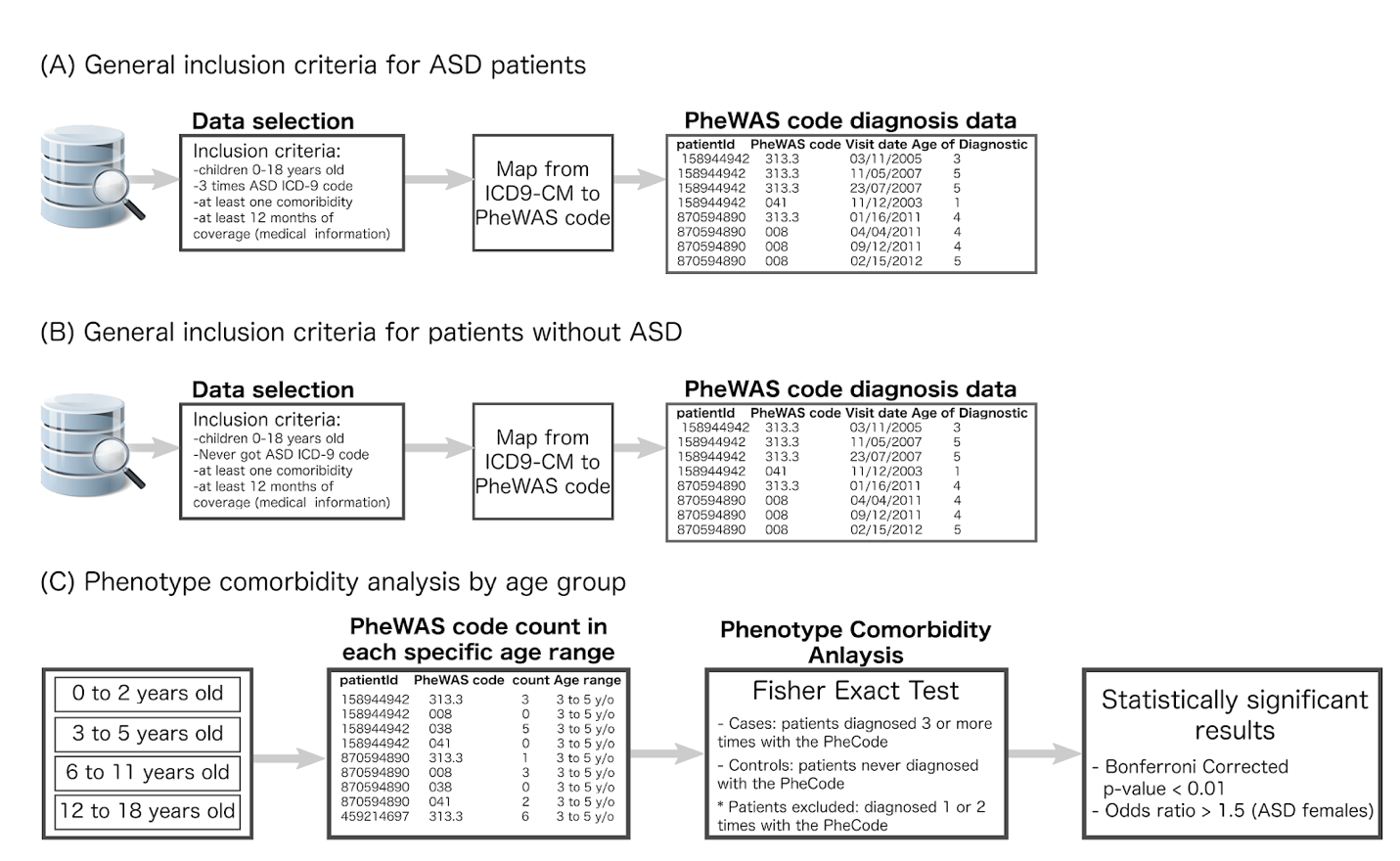
*

*TableS1. Phenotype categories that are statistically significant (corrected p-value < 0.01) in the claims dataset. Results for the PheWAS comparing females with ASD vs males with ASD (OR > 1.5). For each phenotype category, we presented the OR, confidence interval, p-value adjusted by Bonferroni, number of females and males with ASD that presented the phenotype category, the number of females and males with ASD that did not present the phenotype category and the number of females and males with ASD excluded (those that have been diagnosed with the phenotype category code only 1 or 2 times)*

| Age Group | Phenotype Category | OR | 95% Conf.  Interval | p-value (adjusted) | Total patients with the phecode | Female with the phecode | Male with the phecode | Total patients without the phenotype | Female without the phenotype | Male without the phenotype | Total patients with the phenotype excluded | Females with the phenotype excluded | Males with the phenotype excluded |
| --- | --- | --- | --- | --- | --- | --- | --- | --- | --- | --- | --- | --- | --- |
| 0 to 2 | Other specified congenital anomalies of nervous system | 3.386 | (2.048, 5.588) | 1.71E-03 | 70 | 34 | 36 | 7274 | 1586 | 5688 | 103 | 33 | 70 |
| 0 to 2 | Failure to thrive (childhood) | 2.499 | (1.807, 3.438) | 2.85E-05 | 173 | 70 | 103 | 7011 | 1499 | 5512 | 263 | 84 | 179 |
| 0 to 2 | Symptoms concerning nutrition, metabolism, and development | 1.708 | (1.375, 2.115) | 1.61E-03 | 441 | 139 | 302 | 6375 | 1353 | 5022 | 631 | 161 | 470 |
| 3 to 5 | Other cerebral degenerations | 12.543 | (5.481, 32.206) | 1.27E-08 | 33 | 25 | 8 | 18623 | 3713 | 14910 | 36 | 15 | 21 |
| 3 to 5 | Other specified congenital anomalies of nervous system | 2.991 | (2.109, 4.223) | 1.04E-06 | 144 | 61 | 83 | 18299 | 3609 | 14690 | 249 | 83 | 166 |
| 3 to 5 | Epilepsy, recurrent seizures, convulsions | 2.126 | (1.653, 2.719) | 8.39E-06 | 300 | 103 | 197 | 18033 | 3560 | 14473 | 359 | 90 | 269 |
| 3 to 5 | Epilepsy | 2.070 | (1.542, 2.759) | 1.68E-03 | 222 | 75 | 147 | 18202 | 3599 | 14603 | 268 | 79 | 189 |
| 3 to 5 | Chromosomal anomalies | 2.033 | (1.544, 2.66) | 7.31E-04 | 255 | 85 | 170 | 18156 | 3584/ | 14572 | 281 | 84 | 197 |
| 3 to 5 | Partial epilepsy | 2.013 | (1.518, 2.65) | 1.56E-03 | 244 | 81 | 163 | 1819 | 3603 | 14593 | 252 | 69 | 183 |
| 3 to 5 | Strabismus (not specified as paralytic) | 1.936 | (1.586, 2.357) | 2.00E-07 | 492 | 157 | 335 | 17351 | 3381 | 13970 | 849 | 215 | 634 |
| 3 to 5 | Convulsions | 1.916 | (1.618, 2.263) | 1.52E-10 | 698 | 219 | 479 | 16796 | 3236 | 13560 | 1198 | 298 | 900 |
| 3 to 5 | Constipation | 1.744 | (1.463, 2.071) | 1.12E-06 | 677 | 199 | 478 | 16125 | 3108 | 13017 | 1890 | 446 | 1444 |
| 6 to 11 | Other cerebral degenerations | 20.083 | (8.734, 53.75) | 1.27E-14 | 40 | 33 | 7 | 35920 | 6828 | 29092 | 53 | 16 | 37 |
| 6 to 11 | Dementias | 15.307 | (5.476, 52.777) | 2.27E-06 | 23 | 18 | 5 | 35957 | 6845 | 29112 | 33 | 14 | 19 |
| 6 to 11 | Chromosomal anomalies | 2.562 | (2.144, 3.055) | 6.84E-21 | 564 | 209 | 355 | 35024 | 6545 | 28479 | 425 | 123 | 302 |
| 6 to 11 | Epilepsy, recurrent seizures, convulsions | 2.178 | (1.86, 2.545) | 6.49E-18 | 755 | 251 | 504 | 34365 | 6396 | 27969 | 893 | 230 | 663 |
| 6 to 11 | Other specified congenital anomalies of nervous system | 2.174 | (1.666, 2.821) | 1.96E-05 | 268 | 90 | 178 | 35421 | 6683 | 28738 | 324 | 104 | 220 |
| 6 to 11 | Other and unspecified congenital anomalies | 2.163 | (1.614, 2.877) | 3.51E-04 | 224 | 75 | 149 | 35427 | 6688 | 28739 | 362 | 114 | 248 |
| 6 to 11 | Epilepsy | 2.122 | (1.749, 2.566) | 9.72E-11 | 508 | 167 | 341 | 34914 | 6547 | 28367 | 591 | 163 | 428 |
| 6 to 11 | Partial epilepsy | 2.037 | (1.726, 2.397) | 1.86E-13 | 704 | 225 | 479 | 34737 | 6510 | 28227 | 572 | 142 | 430 |
| 6 to 11 | Generalized convulsive epilepsy | 1.999 | (1.581, 2.516) | 1.71E-05 | 353 | 112 | 241 | 35233 | 6645 | 28588 | 427 | 120 | 307 |
| 6 to 11 | Infantile cerebral palsy | 1.983 | (1.611, 2.432) | 2.48E-07 | 450 | 142 | 308 | 35318 | 6662 | 28656 | 245 | 73 | 172 |
| 6 to 11 | Intellectual disability | 1.934 | (1.651, 2.259) | 1.55E-12 | 789 | 242 | 547 | 34247 | 6377 | 27870 | 977 | 258 | 719 |
| 6 to 11 | Strabismus (not specified as paralytic) | 1.834 | (1.599, 2.1) | 3.02E-14 | 1077 | 316 | 761 | 33183 | 6126 | 27057 | 1753 | 435 | 1318 |
| 6 to 11 | Convulsions | 1.791 | (1.589, 2.017) | 1.40E-17 | 1437 | 415 | 1022 | 32610 | 6026 | 26584 | 1966 | 436 | 1530 |
| 6 to 11 | Abdominal pain | 1.741 | (1.525, 1.985) | 1.43E-12 | 1182 | 335 | 847 | 30531 | 5651 | 24880 | 4300 | 891 | 3409 |
| 6 to 11 | GERD | 1.721 | (1.436, 2.056) | 8.80E-06 | 628 | 179 | 449 | 34209 | 6433 | 27776 | 1176 | 265 | 911 |
| 6 to 11 | Fever of unknown origin | 1.690 | (1.489, 1.916) | 2.79E-12 | 1328 | 365 | 963 | 28138 | 5154 | 22984 | 6547 | 1358 | 5189 |
| 6 to 11 | Constipation | 1.632 | (1.448, 1.838) | 5.35E-12 | 1509 | 404 | 1105 | 30996 | 5672 | 25324 | 3508 | 801 | 2707 |
| 12 to 18 | Dementias | 15.899 | (4.289, 87.753) | 1.84E-03 | 15 | 12 | 3 | 28385 | 5705 | 22680 | 56 | 17 | 39 |
| 12 to 18 | Other cerebral degenerations | 14.238 | (5.984, 39.011) | 4.77E-09 | 32 | 25 | 7 | 28385 | 5691 | 22694 | 39 | 18 | 21 |
| 12 to 18 | Other and unspecified congenital anomalies | 2.542 | (1.867, 3.444) | 6.71E-06 | 188 | 73 | 115 | 28054 | 5605 | 22449 | 214 | 56 | 158 |
| 12 to 18 | Dehydration | 2.499 | (1.743, 3.554) | 7.67E-04 | 141 | 54 | 87 | 27792 | 5530 | 22262 | 523 | 150 | 373 |
| 12 to 18 | Chromosomal anomalies | 2.482 | (2.009, 3.059) | 1.70E-13 | 397 | 151 | 246 | 27784 | 5509 | 22275 | 275 | 74 | 201 |
| 12 to 18 | Insomnia | 1.964 | (1.499, 2.556) | 1.58E-03 | 265 | 87 | 178 | 27355 | 5452 | 21903 | 836 | 195 | 641 |
| 12 to 18 | Strabismus (not specified as paralytic) | 1.894 | (1.539, 2.323) | 3.42E-06 | 451 | 143 | 308 | 26877 | 5291 | 21586 | 1128 | 300 | 828 |
| 12 to 18 | Epilepsy | 1.866 | (1.51, 2.298) | 1.67E-05 | 435 | 137 | 298 | 27476 | 5430 | 22046 | 545 | 167 | 378 |
| 12 to 18 | Infantile cerebral palsy | 1.817 | (1.456, 2.257) | 2.60E-04 | 402 | 125 | 277 | 27799 | 5531 | 22268 | 255 | 78 | 177 |
| 12 to 18 | Intellectual disability | 1.783 | (1.568, 2.023) | 5.97E-15 | 1239 | 372 | 867 | 26103 | 5064 | 21039 | 1114 | 298 | 816 |
| 12 to 18 | Developmental delays and disorders | 1.745 | (1.558, 1.952) | 3.93E-18 | 1632 | 481 | 1151 | 24696 | 4772 | 19924 | 2128 | 481 | 1647 |
| 12 to 18 | Muscle weakness | 1.706 | (1.377, 2.105) | 1.73E-03 | 439 | 131 | 308 | 27797 | 5546 | 22251 | 220 | 57 | 163 |
| 12 to 18 | Epilepsy, recurrent seizures, convulsions | 1.696 | (1.455, 1.973) | 3.84E-08 | 866 | 254 | 612 | 26680 | 5245 | 21435 | 910 | 235 | 675 |
| 12 to 18 | Lack of normal physiological development, unspecified | 1.671 | (1.404, 1.983) | 1.60E-05 | 677 | 196 | 481 | 26507 | 5196 | 21311 | 1272 | 342 | 930 |
| 12 to 18 | Dysthymic disorder | 1.659 | (1.393, 1.971) | 2.65E-05 | 670 | 194 | 476 | 27174 | 5358 | 21816 | 612 | 182 | 430 |
| 12 to 18 | Myopia | 1.637 | (1.325, 2.014) | 9.28E-03 | 460 | 133 | 327 | 25450 | 5065 | 20385 | 2546 | 536 | 2010 |
| 12 to 18 | Urinary incontinence | 1.627 | (1.376, 1.918) | 2.41E-05 | 737 | 211 | 526 | 27038 | 5348 | 21690 | 681 | 175 | 506 |
| 12 to 18 | Partial epilepsy | 1.598 | (1.349, 1.887) | 1.22E-04 | 724 | 205 | 519 | 27195 | 5390 | 21805 | 537 | 139 | 398 |

*TableS2. Phenotype categories that are statistically significant (corrected p-value < 0.01) in the claims dataset. Results for the PheWAS comparing females with ASD vs females without ASD (OR > 1.5). For each phenotype category, we presented the OR, confidence interval, p-value adjusted by Bonferroni, number of females with and without ASD that presented the phenotype category, the number of females with and without ASD that did not present the phenotype category and the number of females with and without ASD excluded (those that have been diagnosed with the phenotype category code only 1 or 2 times)*

| Age Group | Phenotype Category | OR | 95% Conf.  Interval | p-value (adjusted) | Total patients with the phecode | Female with ASD with the phecode | Females without ASD with the phecode | Total patients without the phenotype | Female with ASD without the phenotype | Females without ASD without the phenotype | Total patients with the phenotype excluded | Female with ASD with the phenotype excluded | Females without ASD with the phenotype excluded |
| --- | --- | --- | --- | --- | --- | --- | --- | --- | --- | --- | --- | --- | --- |
| 0 to 2 | Other specified congenital anomalies of nervous system | 12.016 | (8.258, 16.941) | 1.75E-21 | 1401 | 34 | 1367 | 767821 | 1586 | 766235 | 2857 | 33 | 2824 |
| 0 to 2 | Failure to thrive (childhood) | 4.498 | (3.484, 5.724) | 3.06E-20 | 7762 | 70 | 7692 | 742330 | 1499 | 740831 | 21987 | 84 | 21903 |
| 0 to 2 | Symptoms concerning nutrition, metabolism, and development | 4.740 | (3.949, 5.652) | 4.13E-43 | 15144 | 139 | 15005 | 693671 | 1353 | 692318 | 63264 | 161 | 63103 |
| 3 to 5 | Other cerebral degenerations | 72.042 | (44.257, 113.241) | 6.82E-33 | 117 | 25 | 92 | 988109 | 3713 | 984396 | 129 | 15 | 114 |
| 3 to 5 | Other specified congenital anomalies of nervous system | 24.262 | (18.309, 31.617) | 1.54E-56 | 746 | 61 | 685 | 986479 | 3609 | 982870 | 1130 | 83 | 104) |
| 3 to 5 | Epilepsy, recurrent seizures, convulsions | 34.544 | (27.822, 42.634) | 9.39E-111 | 925 | 103 | 822 | 985913 | 3560 | 982353 | 1517 | 90 | 1427 |
| 3 to 5 | Epilepsy | 32.671 | (25.301, 41.628) | 1.11E-78 | 702 | 75 | 627 | 986599 | 3599 | 983000 | 1054 | 79 | 975 |
| 3 to 5 | Chromosomal anomalies | 15.010 | (11.892, 18.731) | 4.37E-63 | 1637 | 85 | 1552 | 985819 | 3584 | 982235 | 899 | 84 | 815 |
| 3 to 5 | Partial epilepsy | 31.128 | (24.352, 39.279) | 1.67E-83 | 791 | 81 | 710 | 986645 | 3603 | 983042 | 919 | 69 | 850 |
| 3 to 5 | Strabismus (not specified as paralytic) | 4.958 | (4.192, 5.829) | 3.15E-52 | 9142 | 157 | 8985 | 962724 | 3381 | 959343 | 16489 | 215 | 16274 |
| 3 to 5 | Convulsions | 22.899 | (19.772, 26.377) | 1.07E-199 | 3105 | 219 | 2886 | 979109 | 3236 | 975873 | 6141 | 298 | 5843 |
| 3 to 5 | Constipation | 6.715 | (5.78, 7.765) | 2.60E-87 | 8961 | 199 | 8762 | 922013 | 3108 | 918905 | 57381 | 446 | 56935 |
| 6 to 11 | Other cerebral degenerations | 50.250 | (33.36, 73.628) | 2.92E-39 | 187 | 33 | 154 | 1608563 | 6828 | 1601735 | 190 | 16 | 174 |
| 6 to 11 | Dementias | 120.020 | (64.072, 217.684) | 2.18E-26 | 53 | 18 | 35 | 1608810 | 6845 | 1601965 | 77 | 14 | 63 |
| 6 to 11 | Chromosomal anomalies | 25.225 | (21.715, 29.167) | 2.90E-199 | 2234 | 209 | 2025 | 1605322 | 6545 | 1598777 | 1384 | 123 | 1261 |
| 6 to 11 | Epilepsy, recurrent seizures, convulsions | 33.370 | (29.055, 38.152) | 4.12E-267 | 2129 | 251 | 1878 | 1603360 | 6396 | 1596964 | 3451 | 230 | 3221 |
| 6 to 11 | Other specified congenital anomalies of nervous system | 24.521 | (19.482, 30.516) | 2.48E-84 | 969 | 90 | 879 | 1606402 | 6683 | 1599719 | 1569 | 104 | 1465 |
| 6 to 11 | Other and unspecified congenital anomalies | 18.506 | (14.415, 23.439) | 9.73E-62 | 1044 | 75 | 969 | 1605767 | 6688 | 1599079 | 2129 | 114 | 2015 |
| 6 to 11 | Epilepsy | 23.497 | (19.877, 27.574) | 1.76E-154 | 1903 | 167 | 1736 | 1604742 | 6547 | 1598195 | 2295 | 163 | 2132 |
| 6 to 11 | Partial epilepsy | 27.776 | (24.032, 31.934) | 4.16E-223 | 2214 | 225 | 1989 | 1604424 | 6510 | 1597914 | 2302 | 142 | 2160 |
| 6 to 11 | Generalized convulsive epilepsy | 31.497 | (25.573, 38.416) | 2.55E-116 | 968 | 112 | 856 | 1606212 | 6645 | 1599567 | 1760 | 120 | 1640 |
| 6 to 11 | Infantile cerebral palsy | 16.813 | (14.056, 19.978) | 1.65E-112 | 2169 | 142 | 2027 | 1605787 | 6662 | 1599125 | 984 | 73 | 911 |
| 6 to 11 | Intellectual disability | 131.481 | (111.943, 153.279) | 0.00E+00 | 705 | 242 | 463 | 1607179 | 6377 | 1600802 | 1056 | 258 | 798 |
| 6 to 11 | Strabismus (not specified as paralytic) | 5.306 | (4.718, 5.948) | 1.92E-113 | 15420 | 316 | 15104 | 1559538 | 6126 | 1553412 | 33982 | 435 | 33547 |
| 6 to 11 | Convulsions | 23.617 | (21.217, 26.182) | 0.00E+00 | 5054 | 415 | 4639 | 1595805 | 6026 | 1589779 | 8081 | 436 | 7645 |
| 6 to 11 | Abdominal pain | 2.056 | (1.835, 2.297) | 1.37E-27 | 40256 | 335 | 39921 | 1390049 | 5651 | 1384398 | 178635 | 891 | 177744 |
| 6 to 11 | GERD | 7.853 | (6.715, 9.133) | 1.54E-89 | 5745 | 179 | 5566 | 1577231 | 6433 | 1570798 | 25964 | 265 | 25699 |
| 6 to 11 | Fever of unknown origin | 2.505 | (2.245, 2.787) | 5.65E-47 | 37440 | 365 | 37075 | 1316253 | 5154 | 1311099 | 255247 | 1358 | 253889 |
| 6 to 11 | Constipation | 7.785 | (7.012, 8.625) | 1.01E-199 | 14196 | 404 | 13792 | 1513131 | 5672 | 1507459 | 81613 | 801 | 80812 |
| 12 to 18 | Dementias | 82.438 | (39.601, 158.28) | 1.49E-15 | 56 | 12 | 44 | 1730447 | 5705 | 1724742 | 179 | 17 | 162 |
| 12 to 18 | Other cerebral degenerations | 54.069 | (33.839, 83.481) | 2.72E-30 | 165 | 25 | 140 | 1730292 | 5691 | 1724601 | 225 | 18 | 207 |
| 12 to 18 | Other and unspecified congenital anomalies | 28.540 | (22.088, 36.367) | 6.88E-73 | 859 | 73 | 786 | 1727579 | 5605 | 1721974 | 2244 | 56 | 2188 |
| 12 to 18 | Dehydration | 4.604 | (3.448, 6.03) | 6.73E-16 | 3651 | 54 | 3597 | 1701634 | 5530 | 1696104 | 25397 | 150 | 25247 |
| 12 to 18 | Chromosomal anomalies | 27.246 | (22.863, 32.274) | 4.72E-149 | 1884 | 151 | 1733 | 1727481 | 5509 | 1721972 | 1317 | 74 | 1243 |
| 12 to 18 | Insomnia | 10.151 | (8.087, 12.587) | 1.53E-51 | 2769 | 87 | 2682 | 1711087 | 5452 | 1705635 | 16826 | 195 | 16631 |
| 12 to 18 | Strabismus (not specified as paralytic) | 8.487 | (7.122, 10.042) | 1.25E-75 | 5553 | 143 | 5410 | 1704204 | 5291 | 1698913 | 20925 | 300 | 20625 |
| 12 to 18 | Epilepsy | 25.823 | (21.486, 30.794) | 3.00E-132 | 1819 | 137 | 1682 | 1725956 | 5430 | 1720526 | 2907 | 167 | 2740 |
| 12 to 18 | Infantile cerebral palsy | 21.134 | (17.458, 25.391) | 1.31E-110 | 1966 | 125 | 1841 | 1727564 | 5531 | 1722033 | 1152 | 78 | 1074 |
| 12 to 18 | Intellectual disability | 162.890 | (141.49, 184.347) | 0.00E+00 | 1152 | 372 | 780 | 1728071 | 5064 | 1723007 | 1459 | 298 | 1161 |
| 12 to 18 | Developmental delays and disorders | 76.338 | (68.867, 84.751) | 0.00E+00 | 2749 | 481 | 2268 | 1722668 | 4772 | 1717896 | 5265 | 481 | 4784 |
| 12 to 18 | Muscle weakness | 3.355 | (2.797, 3.994) | 3.23E-27 | 12151 | 131 | 12020 | 1712870 | 5546 | 1707324 | 5661 | 57 | 5604 |
| 12 to 18 | Epilepsy, recurrent seizures, convulsions | 30.268 | (26.447, 34.559) | 1.87E-261 | 3002 | 254 | 2748 | 1722683 | 5245 | 1717438 | 4997 | 235 | 4762 |
| 12 to 18 | Lack of normal physiological development, unspecified | 71.896 | (61.121, 83.963) | 6.46E-269 | 1100 | 196 | 904 | 1726008 | 5196 | 1720812 | 3574 | 342 | 3232 |
| 12 to 18 | Dysthymic disorder | 4.532 | (3.903, 5.236) | 2.84E-59 | 13754 | 194 | 13560 | 1702549 | 5358 | 1697191 | 14379 | 182 | 14197 |
| 12 to 18 | Myopia | 1.765 | (1.474, 2.098) | 5.33E-06 | 23166 | 133 | 23033 | 1553254 | 5065 | 1548189 | 154262 | 536 | 153726 |
| 12 to 18 | Urinary incontinence | 24.359 | (21.014, 28.085) | 6.61E-199 | 2988 | 211 | 2777 | 1719138 | 5348 | 1713790 | 8556 | 175 | 8381 |
| 12 to 18 | Partial epilepsy | 28.837 | (24.812, 33.374) | 4.01E-207 | 2474 | 205 | 2269 | 1725396 | 5390 | 1720006 | 2812 | 139 | 2673 |

*TableS3. Excluded phenotype categories per age group: phenotype categories that were more likely to be present in females with ASD, as compared to males with ASD and females without ASD, and phenotype categories more likely to be present in females without ASD as compared to males without ASD.*

| Age Group | Excluded phenotype categories |
| --- | --- |
| 0 to 2 years old | Other disease of lung |
|  | Cardiac shunt/ heart septal defect |
| 3 to 5 years old | Precocious sexual development and puberty |
|  | Urinary tract infection |
| 6 to 11 years old | Hypothyroidism non elsewhere classified |
|  | Precocious sexual development and puberty |
|  | Urinary tract infection |
|  | Dysuria |
|  | Kyphoscoliosis and scoliosis |
| 12 to 18 years old | Hypothyroidism NOS |
|  | Chronic lymphocytic thyroiditis |
|  | Anxiety disorder |
|  | Major depressive disorder |
|  | Depression |
|  | Anxiety, phobic and dissociative disorders |
|  | Agoraphobia, social phobia, and panic disorder |
|  | Generalized anxiety disorder |
|  | Eating disorder |
|  | Posttraumatic stress disorder |
|  | Otalgia |
|  | Constipation |
|  | Urinary tract infection |
|  | Hirsutism |
|  | Kyphoscoliosis and scoliosis |
|  | Syncope and collapse |
|  | Dizziness and giddiness (Light-headedness and vertigo) |
|  | Malaise and fatigue |
|  | Abnormal weight gain |
|  | Nausea and vomiting |
|  | Dysuria |
|  | Abdominal pain |
|  | Suicidal ideation |

*
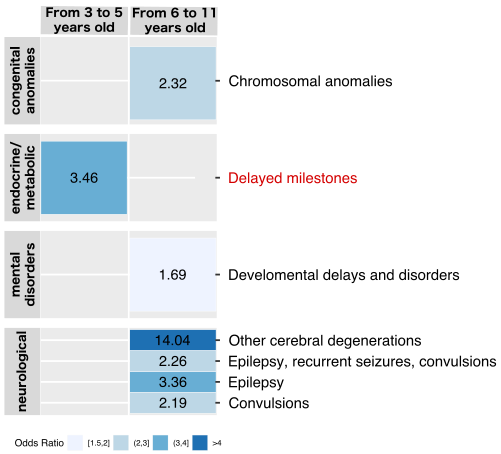
*

*FigS2. Comorbidities more likely to appear in females with ASD in the pediatric hospital by age groups. This heatmap represents the comorbidity phenotypic categories in the different age groups. The Y-axis on the left represents the phenotype categories, the Y-axis on the right represents the specific phenotypes. The phenotypes in red represent those that have not been found as statistically significant in the claims dataset analysis. The X-axis represents the four different age groups. The cell color is related to the odds ratio when comparing ASD females versus ASD males.*

*TableS4. Phenotype categories that are statistically significant (corrected p-value < 0.01) in the pediatric hospital dataset. Results for the PheWAS comparing females with ASD vs males with ASD (OR > 1.5). For each phenotype category, we presented the OR, confidence interval, p-value adjusted by Bonferroni, number of females and males with ASD that presented the phenotype category, the number of females and males with ASD that did not present the phenotype category and the number of females and males with ASD excluded (those that have been diagnosed with the phenotype category code only 1 or 2 times)*

| Age Group | Phenotype Category | OR | 95% Conf.  Interval | p-value (adjusted) | Total patients with the phecode | Female with the phecode | Male with the phecode | Total patients without the phenotype | Female without the phenotype | Male without the phenotype | Total patients with the phenotype excluded | Females with the phenotype excluded | Males with the phenotype excluded |
| --- | --- | --- | --- | --- | --- | --- | --- | --- | --- | --- | --- | --- | --- |
| 3 to 5 | Delayed milestones | 3.458 | (2.029, 5.859) | 2.61E-03 | 66 | 30 | 36 | 1917 | 372 | 1545 | 209 | 51 | 158 |
| 6 to 11 | Other cerebral degenerations | 14.035 | (4.387, 58.8) | 2.88E-04 | 18 | 14 | 4 | 2864 | 571 | 2293 | 12 | 4 | 8 |
| 6 to 11 | Epilepsy | 3.363 | (2.196, 5.125) | 1.87E-05 | 102 | 45 | 57 | 2689 | 511 | 2178 | 103 | 33 | 70 |
| 6 to 11 | Chromosomal anomalies | 2.320 | (1.698, 3.151) | 1.28E-04 | 216 | 75 | 141 | 2499 | 466 | 2033 | 179 | 48 | 131 |
| 6 to 11 | Epilepsy, recurrent seizures, convulsions | 2.263 | (1.577, 3.219) | 9.31E-03 | 159 | 55 | 104 | 2556 | 484 | 2072 | 179 | 50 | 129 |
| 6 to 11 | Convulsions | 2.190 | (1.61, 2.961) | 5.08E-04 | 230 | 77 | 153 | 2264 | 423 | 1841 | 400 | 89 | 311 |
| 6 to 11 | Developmental delays and disorders | 1.694 | (1.357, 2.111) | 2.55E-03 | 725 | 184 | 541 | 1519 | 254 | 1265 | 650 | 151 | 499 |

*TableS5. Phenotype categories that are statistically significant (corrected p-value < 0.01) in the pediatric hospital dataset. Results for the PheWAS comparing females with ASD vs females without ASD (OR > 1.5). For each phenotype category, we presented the OR, confidence interval, p-value adjusted by Bonferroni, number of females with and without ASD that presented the phenotype category, the number of females with and without ASD that did not present the phenotype category and the number of females with and without ASD excluded (those that have been diagnosed with the phenotype category code only 1 or 2 times)*

| Age Group | Phenotype Category | OR | 95% Conf.  Interval | p-value (adjusted) | Total patients with the phecode | Female with ASD with the phecode | Females without ASD with the phecode | Total patients without the phenotype | Female with ASD without the phenotype | Females without ASD without the phenotype | Total patients with the phenotype excluded | Female with ASD with the phenotype excluded | Females without ASD with the phenotype excluded |
| --- | --- | --- | --- | --- | --- | --- | --- | --- | --- | --- | --- | --- | --- |
| 3 to 5 | Delayed milestones | 2.15E-13 | 107 | 30 | 77 | 8881 | 372 | 8509 | 220 | 51 | 169 | 2.15E-13 | 107 |
| 6 to 11 | Other cerebral degenerations | 1.08E-05 | 41 | 14 | 27 | 11395 | 571 | 10824 | 14 | 4 | 10 | 1.08E-05 | 41 |
| 6 to 11 | Epilepsy | 2.29E-13 | 220 | 45 | 175 | 11058 | 511 | 10547 | 172 | 33 | 139 | 2.29E-13 | 220 |
| 6 to 11 | Chromosomal anomalies | 4.58E-25 | 362 | 75 | 287 | 10919 | 466 | 10453 | 169 | 48 | 121 | 4.58E-25 | 362 |
| 6 to 11 | Epilepsy, recurrent seizures, convulsions | 5.95E-17 | 274 | 55 | 219 | 10942 | 484 | 10458 | 234 | 50 | 184 | 5.95E-17 | 274 |
| 6 to 11 | Convulsions | 8.21E-23 | 434 | 77 | 357 | 10571 | 423 | 10148 | 445 | 89 | 356 | 8.21E-23 | 434 |
| 6 to 11 | Developmental delays and disorders | 6.68E-91 | 823 | 184 | 639 | 9948 | 254 | 9694 | 679 | 151 | 528 | 6.68E-91 | 823 |

*TableS6. Prevalence of epilepsy and intellectual disabilities in ASD and non-ASD individuals across the four different age groups.*

|  | Group | # total patients | Epilepsy | ID | Epilepsy AND ID |
| --- | --- | --- | --- | --- | --- |
| From 0 to 2 | ASD females | 1,653 | 31 (1.88%) | 336 (20.33%) | 16 (0.97%) |
|  | ASD males | 5,794 | 73 (1.26%) | 998 (17.22%) | 32 (0.55%) |
|  | non-ASD females | 770,426 | 1,234 (0.16%) | 5,782 (0.75%) | 337 (0.04%) |
|  | non-ASD males | 682,969 | 900 (0.13%) | 6,204 (0.91%) | 175 (0.03%) |
| From 3 to 5 | ASD females | 3,753 | 167 (4.45%) | 796 (21.21%) | 93 (2.48%) |
|  | ASD males | 14,939 | 356 (2.38%) | 2,904 (19.44%) | 166 (1.11%) |
|  | non-ASD females | 984,602 | 1,659 (0.17%) | 4,397 (0.45%) | 347 (0.04%) |
|  | non-ASD males | 966,827 | 1,739 (0.18%) | 7,642 (0.79%) | 321 (0.03%) |
| From 6 to 11 | ASD females | 6,877 | 441 (6.41%) | 1,053 (15.31%) | 189 (2.75%) |
|  | ASD males | 29,136 | 974(3.34%) | 3,341 (11.47%) | 296 (1.02%) |
|  | non-ASD females | 1,602,063 | 4,479 (0.28%) | 4,320 (0.27%) | 612 (0.04%) |
|  | non-ASD males | 1,624,984 | 4,656 (0.29%) | 7,278 (0.45%) | 545 (0.03%) |
| From 12 to 18 | ASD females | 5,734 | 447 (7.80%) | 743 (12.96%) | 187 (3.26%) |
|  | ASD males | 22,722 | 1,104 (4.86%) | 1,848 (8.13%) | 319 (1.40%) |
|  | non-ASD females | 1,724,948 | 5,809 (0.34%) | 2,861 (0.17%) | 514 (0.03%) |
|  | non-ASD males | 1,733,307 | 5,508 (0.32%) | 3,628 (0.21%) | 488 (0.03%) |

**Supplemental 1: multiple testing correction**

Since we were analyzing more than 1,000 PheWAS categories in all age groups under study, the Bonferroni correction was applied on the ranked list to correct for multiple testing. The corrected p-value applied was 0.01 for all age groups. For each age group, the cut-off p-value is different, since we have different numbers of phenotypic categories analyzed. The final p-value is 0.01/total Phenotype categories analyzed. The specific cut-off applied to each age group when comparing ASD females vs ASD males was the next:

- 0 to 2 y/o: 0.01/1295 = 7.72 x 10-6
- 3 to 5 y/o: 0.01/1520 = 6.58 x 10-6
- 6 to 11 y/o: 0.01/1661 = 6.02 x 10-6
- 12 to 18 y/o: 0.01/1707 = 5.86 x 10-6
